# Supplementary material for: Transport and Retention of Poly(Acrylic Acid-co-Maleic Acid) Coated Magnetite Nanoparticles in Porous Media: Effect of Input Concentration, Ionic Strength and Grain Size
Source: Nanomaterials (Basel). 2022 May 2;12(9):1536. doi: 10.3390/nano12091536 (PMC9103219; doi:10.3390/nano12091536)
Supplement: Supplementary file 1 [file nanomaterials-12-01536-s001.zip › nanomaterials-1693524-supplementary.pdf]

The supplementary materials

Transport and Retention of Poly(Acrylic Acid-co-Maleic Acid) Coated Magnetite Nanoparticles in Porous Media: Effect of Input Concentration, Ionic Strength and Grain Size

Rawan Mlih [1,2,*], Yan Liang [3], Miaoyue Zhang [4], Etelka Tombácz [5], Roland Bol [1,6] and Erwin Klumpp [1]

1 Institute of Bio- and Geosciences, Agrosphere (IBG–3), Research Centre Juelich (FZJ), 52425 Juelich, Germany; r.bol@fz-juelich.de (R.B.); e.klumpp@fz-juelich.de (E.K.)

2 Institute for Environmental Research, Biology 5, RWTH Aachen University, 52074 Aachen, Germany

3 School of Resources, Environment and Materials, Guangxi University, Nanning 530004, China;
liangyan@gxu.edu.cn

4 School of Environmental Science and Engineering, Sun Yat-sen University, Guangzhou 510006, China; zhangmy53@mail.sysu.edu.cn

5 Soós Ernő Water Technology Research and Development Center, University of Pannonia,
H-8800 Nagykanizsa, Hungary; e.tombacz@chem.u-szeged.hu

6 School of Natural Sciences, Environment Centre Wales, Bangor University, Bangor LL57 2DG, UK

* Correspondence: r.mlih@fz-juelich.de

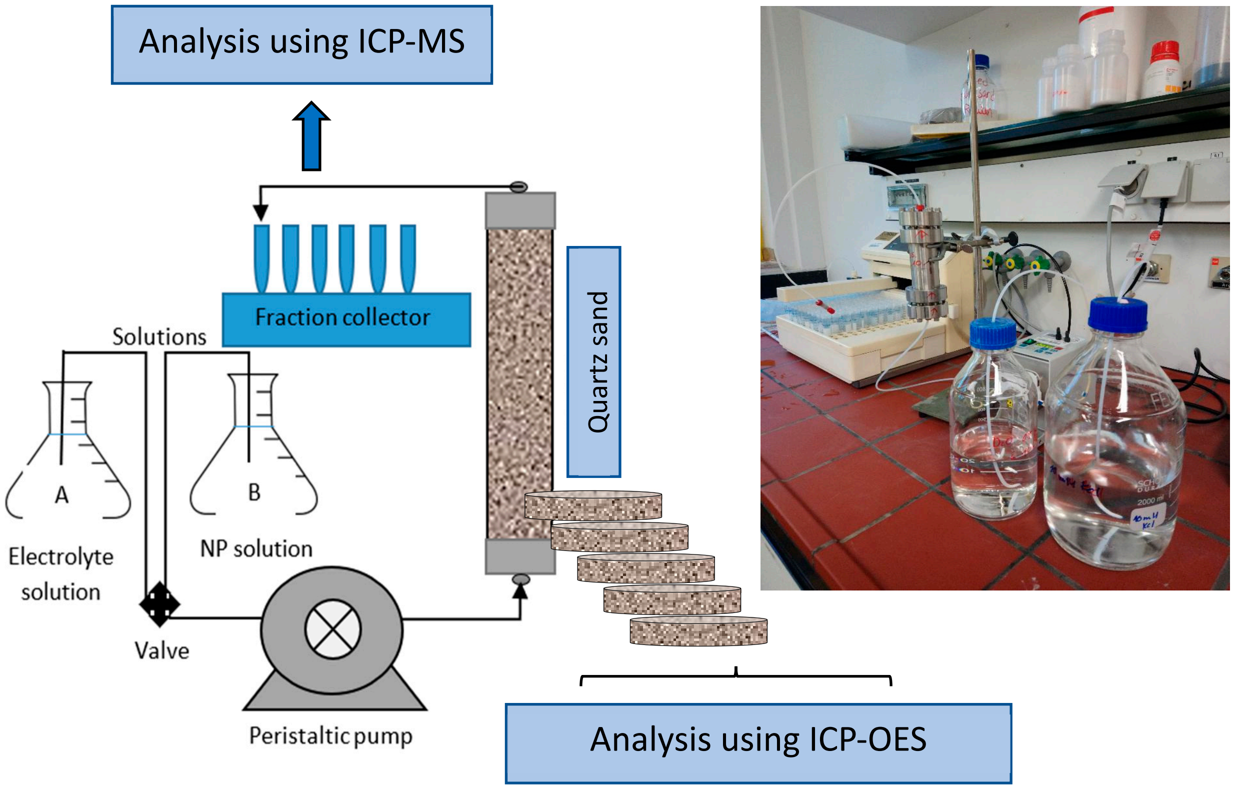

Figure S1.

Figure S1. Schematic diagram of water-saturated column set-up. Tracer, PAM@MNP and electrolyte solutions were separately pumped through the column in an upflow mode. A three-way value was used to switch between the solutions.

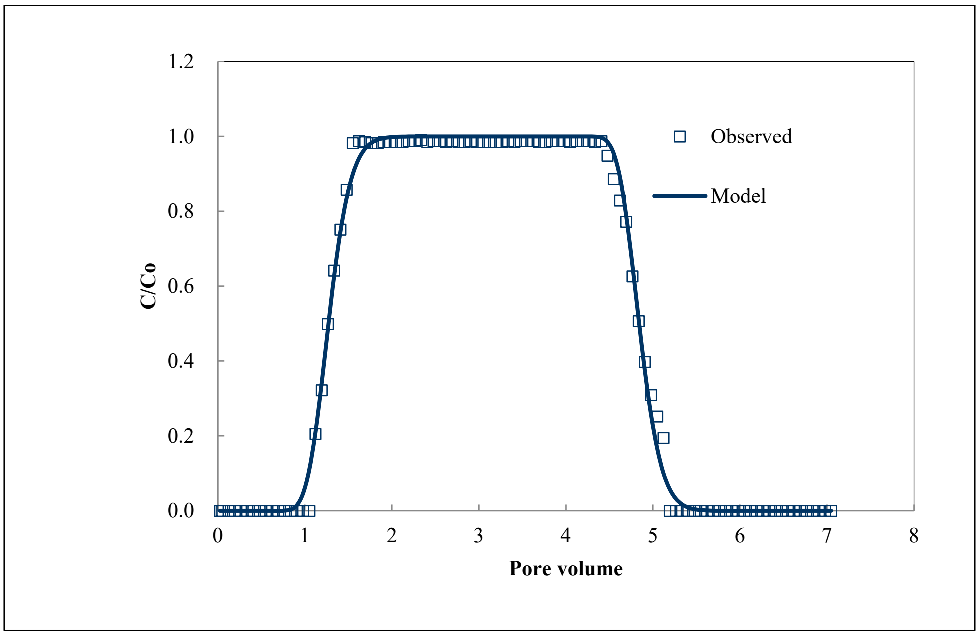

Figure S2. Example of the BTC for D_2O tracer. All BTCs for the tracer for all columns experiments presented the similar shapes.

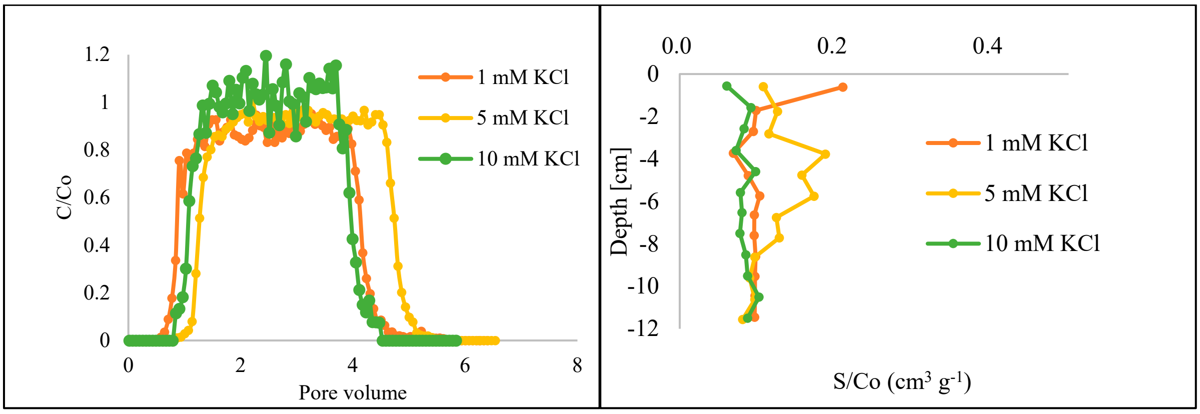

Figure S3. Observed BTCs and RPs for PAM@MNP at 1, 5 and 10 mM. Initial concentration 5 mg L^{-1}, Darcy velocity: 0.29 – 3.0 cm min^{-1}.
